# Environmental sustainability and the limits of healthcare resource allocation

**DOI:** 10.1111/bioe.13395

**Published:** 2025-01-27

**Authors:** James Hart, Sapfo Lignou, Mark Sheehan

**Affiliations:** ^1^ Ethox Centre Nuffield Department for Population Health, University of Oxford Oxford UK; ^2^ NIHR Oxford Biomedical Research Centre Oxford UK

**Keywords:** climate change, environmental sustainability, priority setting, resource allocation

## Abstract

Recent literature has drawn attention to the complex relationship between health care and the environmental crisis. Healthcare systems are significant contributors to climate change and environmental degradation, and the environmental crisis is making our health worse and thus putting more pressure on healthcare systems; our health and the environment are intricately linked. In light of this relationship, we might think that there are no trade‐offs between health and the environment; that healthcare decision‐makers have special responsibilities to the environment; and that environmental values should be included in healthcare resource‐allocation decisions. However, we argue that these claims are mistaken. The environmental crisis involves a wider range of considerations than just health. There is a plurality of reasons to act on the environment; we might do so to protect the natural world, to prevent catastrophes in other parts of the world, or to avert climate war and displacement. Trading‐off between health care and environmental sustainability is thus unavoidable and requires sensitivity to all these reasons. Healthcare decision‐makers are not well placed to be sensitive to these reasons, nor do they have the democratic authority to make such value judgements. Therefore, decisions about environmental sustainability interventions should be made at a ‘higher level’ of resource allocation. Importantly, hospitals have environmental duties but not environmental responsibilities; their job is to provide the best healthcare possible within the constraints given to them, not to choose between health care and other goods.

## 1 ETHICAL RESOURCE ALLOCATION

There are insufficient resources to fund every good intervention. This truism is the fundamental problem of resource allocation, and it requires that allocators trade‐off between different options, including options which are not otherwise opposed. It would thus be helpful to begin by explaining why typical resource allocation decisions are difficult, and the solution that many places have adopted.

First, we start with the recognition that there are no value‐free ways of making resource allocation decisions. This is true in the broader context and in the narrower healthcare context. In the broader context, we must weigh the value of investing in healthcare against the value of investing in education, in justice, in the arts, and so forth. In the narrower healthcare context, we have to weigh the value of treating people now versus later, judge between our obligations to individuals and to the population as a whole, and so forth.

Importantly, there will be significant moral disagreement on how to handle these trade‐offs. Of course, this disagreement needs to be settled, and the manner in which it is settled needs to ensure the solution we come to has legitimacy. To do this, we typically use democratic systems to provide a fair process for everyone to voice their values and perspectives. Thus, when we cannot agree on a substantively correct answer, all stakeholders can at least accept the decision as the result of a process that gives consideration to everyone.

In the broader resource allocation context, these decisions are handled directly by democratic institutions. Elected officials decide how much funding is available to health care compared to education, justice, and the arts.^1^ However, not all decisions can be settled in this way, and resource allocation within health care is such a case. Resource allocation in health care requires too much information, too much expertise, and involves simply too many decisions to be accurately and effectively settled by democratically elected officials, or other wider democratic practices. In response to this problem, many democratic systems pass over such decision‐making to the healthcare sector in various forms. Doing so grants the relevant institutions limited authority to make allocation decisions within certain domains and under certain constraints. For instance, in the United Kingdom, the government passes over decisions on the cost‐effectiveness and value for money of particular treatments to the National Institute for Clinical Excellence (NICE). Local commissioners are then tasked with deciding whether and how it will fund these treatments. Local decision‐making allows for greater sensitivity to the local needs, and trade‐offs relevant to their specific regions. There are, of course, even lower levels of decision‐making (bed allocation, staffing distribution, healthcare professionals' own decisions), but, in this paper, we focus on those bodies (either at the national, local, or hospital level) that make specific funding decisions.^2^

Nevertheless, these decision‐making bodies do not receive their legitimacy solely from the approval of higher level democratic institutions. These bodies are still required to abide by certain decision‐making processes and constraints. These processes provide a coherent structure for decision‐making, aim to promote fairness and consistency in the decision‐making process, include diverse perspectives and values, and ensure that decisions are reasonable and decision‐makers accountable. It is this model of ‘Accountability for Reasonableness’ that ensures these decision‐making bodies are eligible to bear the decision‐making responsibility delegated to them.^3^ In the United Kingdom, this model is operationalised with the use of ‘Ethical Frameworks’.^4^ These frameworks require decision‐makers to carefully consider a wide range of values and factors, whilst also excluding other factors from consideration. For instance, in the United Kingdom, decision‐makers must consider the effects of any decision on health inequalities, whilst they are simultaneously constrained from prioritising care based on personal characteristics such as age, race, or gender.

It is against this background that resource allocation decisions are constrained, and many wider considerations are excluded from the decision‐making process. This includes some health benefits. For instance, the most impactful thing for health a local NHS Trust could do might be to fund anti‐malaria campaigns in LMICs. But this is significantly outside the scope of the Trust's responsibility. It is up to democratically elected governments to decide how much money is given to foreign aid and how much is given to local health needs.

Another useful comparison is with education. Education, and the institutions of education, have significant positive effects on health, and thus investing in education may in fact be cost‐effective at the population level in terms of health effects.^5^ Nevertheless, the primary benefit, or value, of education is not its impact on health, and trade‐offs between health care and education cannot and should not be weighed in entirely health terms. Moreover, we would consider it inappropriate if a hospital gave its money away to schools. It is up to the wider democratic system to decide how much resource goes towards health care and how much goes towards education. Where there is overlapping value, and hospitals believe that it may be cost‐effective to spend more on certain educational programmes, then hospitals can collaborate with education partners and outline the health benefits of education to higher level decision makers, but it is not their responsibility to directly fund education.

With this in view, we must ask whether environmental sustainability fits within the scope of healthcare resource allocation. Is environmental sustainability more like health inequalities or more like education? Do healthcare institutions have the appropriate responsibility, capability, and legitimacy to properly consider environmental factors in their own decision‐making? In our view, the answer is no. At least not in the sense that they have responsibility for primarily health factors.

## 2 THE VALUE(S) OF ENVIRONMENTAL SUSTAINABILITY

The environment, and environmental sustainability, are slightly vague concepts that involve an array of different issues. Rather than define these concepts, we shall rely on the common understanding of these terms involving issues such as climate change, loss of natural habitats, pollution, exhaustion of non‐renewable resources, and so forth. Sometimes we will use more specific examples, in particular climate change as the paradigmatic issue, to make our arguments, but these arguments apply to the whole range of environmental issues and values therein.

Let us take climate change as the paradigmatic example of an environmental issue. Climate change is complex and unpredictable. The effects of healthcare on climate change might be large, but they are hard to quantify. Similarly, the effects of climate change on health will be large, but they too are hard to quantify. As such, we can view climate change as a chaotic black box system. We understand the inputs that contribute to climate change, and we can predict, but only in very broad terms, the outputs of the system. However, we are not able to draw a direct line from singular inputs to singular outputs.

This fact makes the problem of climate change, and our ensuing obligations, much more complex to analyse. It becomes very difficult to identify and quantify the health costs due to climate change of different healthcare interventions or policies. For instance, converting fleets of ambulances to electric vehicles will reduce the quantity of greenhouse gases emitted. This reduction will have a minor (perhaps imperceptible) effect on climate change and thus produce potential future healthcare benefits by reducing air pollution, the chance of extreme‐heat events, and so forth.

We might think that this means framing the problem as a form of trade‐off between acute or immediate care and preventative or public health interventions, albeit preventative interventions with a great deal of uncertainty. We already handle problems with similar features in healthcare decision‐making and resource allocation. We make trade‐offs between acute interventions and uncertain future benefits in the context of policies, for example, intended to reduce the chance of anti‐biotic resistance or in pandemic preparedness measures like stockpiling vaccines.

Nevertheless, we think this framing is wrong in this context. The complexity and unpredictability of climate change does not only make the problem more complex to analyse but it also changes the obligations we have and where those obligations lie. This is especially true in the context of resource allocation.

The first thing to note is that climate change and environmental sustainability are societal problems that involve a wider range of harms than just healthcare harms. The value of environmental sustainability is not entirely (nor even primarily) in terms of health. There are, thus, a plurality of reasons to act on climate change; we might do so to protect the natural world, to prevent catastrophes in other parts of the world, to preserve the world for future generations, to ensure productive and stable economies, or to avert climate war and displacement. In this way, environmental sustainability is much more like the case of education; it overlaps significantly with health, but cannot be reduced to health.

Moreover, the responsibility for climate change is diffused across the entirety of our national and global society. Mitigating the climate contributions of healthcare will mean little if there is a corresponding increase in the climate contributions of say national defence or transportation. This is not to say that the healthcare sector has no duties to the environment or that it is excused from its duties because other sectors are worse. But it does mean that the healthcare sector cannot act unilaterally and that the healthcare sector's environmental obligations cannot be examined in isolation. Whilst healthcare institutions are large employers and large polluters, they remain only a small part of the whole range of national (and international) institutions which share this responsibility altogether.

Both these elements make decision‐making about environmental sustainability much more difficult. First, trade‐offs are much more complex when we have to weigh a greater array of values. How ought we to weigh the benefit of helping protect the natural world, against the clinical benefits of single‐use plastics such as medical gloves? How does a miniscule increase in the chance of climate displacement weigh against the healthcare benefits of transporting patients between hospitals?

Second, making judgements about competing responsibilities and obligations requires having a sense of how much responsibility we have for each factor. Clearly, we do not have responsibility for all foreseeable consequences of healthcare actions; a hospital that saves the life of a billionaire who regularly flies a private jet has no responsibility for these climate consequences. With this in mind, is the healthcare sector responsible for a share of all negative consequences from climate change that it foreseeably contributes to, or only some? If so, which ones? Only the direct health effects? What about secondary health effects, such as the health effects of climate war?

## 3 LIMITED SCOPE

All these questions highlight just how ethically fraught this issue is. Importantly, it is ethically fraught in dimensions that typical healthcare resource allocation questions are not. Of course, that it is ethically fraught does not itself mean environmental factors should be excluded from healthcare resource allocation decision‐making. We need separate reasons to think that the nature of the problem means it falls outside of the scope of healthcare decision‐making.

First, we think there are some practical reasons not to include these considerations in decision‐making. It seems clear to us that decision‐makers in healthcare do not have the necessary evidence or expertise to be sensitive to these kinds of reasons. Members of priority‐setting committees, HTA organisations, and similar bodies are constituted of experts in the health domain. They include clinicians with different specialisms; hospital and wider system managers and support units; experts in health economics, law, and ethics; and lay members with significant familiarity with the healthcare sector. Together these people are proficient at identifying and analysing different considerations and judging difficult trade‐offs in healthcare. They are capable of doing so efficiently, consistently, and fairly.

Nevertheless, in our experience, these committees work much better, when there is consistent and good‐quality evidence and when the scope of considerations are much narrower. When these committees have been tasked with considering non‐health values, or non‐typical health values, as part of their decisions, they have often struggled to come to agreement. For instance, the benefits of Assisted Reproductive Technologies (ARTs) are quite different from typical healthcare benefits,^6^ and they require the consideration of a broader set of incommensurable values. As such, decision‐making about ARTs has been especially difficult, time‐consuming, and inefficient.

It is our view that the consideration of environmental sustainability in resource allocation decisions would further complicate already difficult decisions. The committee does not have the expertise to identify and evaluate different environmental factors and values; it would lack the ability to understand and interpret the evidence; and it seems unlikely that good‐quality evidence of the effects of particular interventions would be readily available.

Second, and perhaps more importantly, there are ‘in‐principle’, ethical reasons not to include environmental considerations in decision‐making. It is simply not the delegated responsibility of hospitals or healthcare decision‐makers to address (or choose how to address) environmental sustainability. As we saw above, decision‐making legitimacy in healthcare resource allocation comes in large part from being given that responsibility by higher‐level democratic institutions. Thus, in so far as decision‐makers in these contexts have not been delegated such responsibility, they do not have the authority to make these trade‐offs.

But this is only a partial answer. Our pursuit is a normative one, so we must also ask whether healthcare decision‐makers *ought* to be delegated such responsibility. Again though, our answer is no. As we saw before, there are many independent and incommensurate reasons we might act on the environment, and many of these reasons are not concerned with health. So, we have two options. We either (a) limit the environmental factors and values that healthcare decision‐makers can act on to those that concern health or (b) we require healthcare decision‐makers to act on a significantly wider set of values. Both of these options strike us as deeply flawed.

Option (a) risks significantly understating the importance of environmental sustainability and distorting the priorities within environmental sustainability efforts. If decision‐makers are only concerned with the health effects, then environmentally sustainable interventions that protect the natural world, or help to protect the economy, or reduce climate catastrophe, displacement, and war, will not be considered at all. This will be especially problematic if decision‐makers are further limited to considering only those health effects that affect their own populations.

Suppose, for instance, that a hospital has two options they can spend their remaining budget on. Either they can convert their fleet of ambulances to electric vehicles or they can spend the extra funds on shifting procurement towards green energy. The first option reduces GHG emissions a little, but significantly improves the air quality in and around the hospital. The second option does not improve air quality, but it does have a much more significant impact on GHG emissions. If we were to take into account all the environmental considerations and the full gamut of reasons to act on the environment, we would conclude that we ought to prioritise procuring green energy. Yet, by limiting the reasons, healthcare allocators can act upon to health, the first option will win out.

Option (b) avoids this problem (in theory at least). If commissioners must consider all the different values, then they will choose to prioritise green energy over electric vehicles and choose the all‐things‐considered better option. Nevertheless, option (b) is perhaps even less tenable than option (a). First, option (b) clearly worsens the practical difficulties of including environmental sustainability in decision‐making. Not only would decision‐makers have to consider and weigh evidence that sits significantly outside of their expertise but they would have to make more trade‐offs between health and non‐health benefits and take into consideration a much wider range of incommensurable values.

As something of an aside, our hospital in the example above is in practice unlikely to be faced with this choice of options. Converting the fleet of ambulances or shifting procurement towards green energy will compete against clear healthcare‐related options: say, reinvigorating the patient liaison services, employing an additional bereavement midwife or updating the ventilator system in the ICU. So, the choice to invest in either of the environmentally sustainable options will come at an opportunity cost of one of the others, where each of the others have very much clearer and specifiable health benefits.

Returning to the initial example, priority setting committees and commissioners are simply not well placed to make these environmental sustainability decisions accurately and legitimately. It would be like giving healthcare decision makers responsibility for funding education as well as health care: not only do they not have the skills to do so accurately, they are also likely to overvalue health care and undervalue education! Of course, one solution to this is to include more people in the resource allocation process. We might include environmental scientists, experts in environment law and ethics, and other environmental stakeholders, and so forth. But it should be obvious that this would be a mess. The range of expertise and the gap in understanding would be far too wide for any effective decision‐making to take place. One of the key reasons for delegating healthcare decision‐making in the first place is that it requires a level of expertise to be able to accurately and fairly weigh difficult trade‐offs in health.

Perhaps despite these complications, we nevertheless ought to include environmental sustainability and environmental experts in our decision‐making processes. But why should we limit ourselves there? Education also matters, and there is significant evidence of the relationship between education and health. So, we ought to include educational experts and stakeholders too. Come to think of it, policing and the justice system matter too, so perhaps we ought to include them too. Although now it looks like this committee has a lot of power over how we allocate resources, so maybe we ought to elect the members of the committee. And, oh look, we have a representative democracy with insufficient expertise in health to make good decisions about health resource allocation. So maybe it would be worthwhile delegating responsibility for healthcare resource allocation decisions to robust decision‐making process within the healthcare sector.

We are being deliberately facetious here, but our intention is merely to show that there is a significant tension between the depth and width of considerations that decision‐makers can incorporate. We can be maximally wide by considering every possible domain, factor, and value in our decisions but in doing so we will lose much of the precision, expertise, and sensitivity required of good decision‐making in health care. Conversely, we can maximise depth of decision‐making on specific issues, but in doing so, miss the bigger picture. This leaves us with two questions: (1) how do we solve this tension and (2) ought we give up some depth in healthcare decision‐making to incorporate environmental sustainability in our decision‐making?

The answer to the first question gives us the answer to the second; we have tiered decision‐making. The highest levels of decision‐making focus on weighing, in the broadest terms, all possible domains—they are maximally wide. They then delegate decision‐making, with financial and other constraints, to other decision‐makers who can focus on depth. Of course, this is broadly the system we already have in place. The government decides on how much funding healthcare receives compared to education and the environment, and so forth, and it sets broad side constraints on how funding can be spent. It then delegates how exactly those funds are spent to decision‐makers with the depth of knowledge to make good decisions within that domain. This does not completely solve the problem, and there can be alignment problems and perverse incentives, and so forth.^7^ but this is the solution many places have settled on, and it works well for the most part.^8^

With all of this in view, it seems clear that decisions weighing the value trade‐offs between health and environmental sustainability clearly sit at the higher level. Good healthcare decision‐making requires depth not width, and there is good reason not to sacrifice some of this depth in order to include environmental considerations, when those considerations can be included at a higher level.

## 4 DUTIES AND RESPONSIBILITY

Now this might all sound like an argument that the healthcare sector has no obligations towards environmental sustainability; that we are defending an ‘anything goes’ mentality when it comes to resource allocation in health care. This is very much not our intention. We simply believe that it is not up to the healthcare sector to decide what its environmental obligations are and how it weighs those obligations against other obligations. Crucially on our view, the healthcare sector is a part of society like any other and so has environmental obligations like any other. What it does not have on our view, are special health‐related responsibilities with respect to the environment. The same is true of individuals in this respect. Individual healthcare practitioners are also citizens and so they have obligations as citizens. But insofar as their roles in healthcare are tied to caring for patients, they do not, by those roles, have special responsibilities to the environment.

It might be helpful here to draw on the subtle, but common language, distinction between duties and responsibilities. A duty is a specific moral or legal obligation, usually regarding some particular action, and it typically constrains an agent's actions by requiring adherence with that obligation in some predetermined way. For instance, a healthcare professional has a duty not to discriminate on the basis of any protected characteristic. This constrains the professional's work in a specific way and does not permit the professional much scope on how to meet the obligation. Conversely, a responsibility is a much broader moral or legal obligation usually regarding some particular domain. It typically sets a broad aim and permits the agent a much wider scope of control about what the obligation entails and how to meet it. For instance, a healthcare professional has responsibility for a patient's care, this permits the professional to consider many different options, to judge between them, and to make decisions about how to best meet the obligation (in concert with the patient).

What we are arguing then is not that the healthcare sector does not have environmental duties, only that it does not have environmental responsibilities. Responsibility for environmental sustainability lies elsewhere. Namely, it lies with higher‐level decision makers who are tasked with weighing health against the environment (and against other considerations like education, social care, and the Arts), deciding how much money to spend on healthcare and on the environment, but also for deciding what environmental duties others have. This includes setting various targets, regulations, constraints, and legal duties that the health care and other sectors must meet in order to fulfil these wider obligations. For instance, they might require that waste is disposed in a more environmentally friendly way or that energy may only be procured from sustainable sources.

In this broad sense, higher‐level decision makers might institute rules about the quantity of GHG emissions that can be released or that transport fleets of state‐funded institutions should be electric.^9^ This might mean that hospitals (and schools) must reduce their carbon footprint by a certain amount or hospitals being required to remain within a specific carbon budget.^10^ In this way, healthcare allocators (like those in education) will still have significant obligations to the environment. But crucially they will not be responsible for judging between health care and the environment, and they will not be weighing the environment in their decision‐making. Instead, they will be merely allocating for health within additional side‐constraints.^11^

We already have such constraints in place throughout health care. Hospitals have obligations to their staff that are encoded in law and that affects the allocation of available resources. Ambulances must meet road safety standards just as any other vehicle does. Individuals might have legal rights that supersede decisions about resource allocation; for instance, patients might have rights to choose where they receive their treatment even if treatment can be provided at lower cost in particular environments. Certain features are excluded from our decisions; someone's job, their social role, and how much tax they pay are excluded from consideration even though they could have effects on overall health.

All of these factors involve value choices and trade‐offs, and they thus influence resource allocation decision‐making in some way. But they are also all choices that involve a much wider range of reasons and values and are thus made outside of the healthcare resource allocation process. It seems clear to us that environmental sustainability also belongs in this category. Ultimately, the healthcare sector ought not to be responsible for deciding how much carbon it is permitted to use just as it ought not to be responsible for deciding how much money it gets to use.

## 5 WHAT CAN HEALTHCARE ACTORS DO?

What we are calling for here then is large systemic change, but one that primarily sits outside of healthcare allocation itself. There is a danger that by focusing on environmental sustainability within our domain, we can miss the bigger picture; by trying to do our part, we can fail to see that the problem is a collective one needing collective change and societal solutions.

Nevertheless, this systemic change does not seem to be forthcoming; political failure has led us to the situation we are in now, so we might wonder whether we can wait for higher‐level decision makers to address these questions in light of the urgency of the impending climate catastrophe. Against this background, it does seem appropriate to ask what healthcare actors can do when higher‐level decision‐makers are failing in their responsibilities. This is a good question, but healthcare decision‐makers taking environmental sustainability decision‐making into their own hands is the wrong answer. Instead, environmentally minded decision‐makers should try to motivate change at the higher level.

As an analogy, some doctors might think that euthanasia ought to be legal (and they might be correct) but, of course, it would be wrong for a doctor to take the law into her own hands and provide euthanasia to patients who wanted it without wider systemic change. Similarly, some doctors might think that a particular war is unjust and ethically reprehensible. For these doctors, there may be ways that they are able to utilise their skills closer to the conflict zone. Importantly, individual doctors in both these cases can, and perhaps should, use their positions to bring these issues to the attention of higher‐level decision‐makers and institute systemic or political change. Healthcare actors are perhaps particularly well placed, for example, to make environmental calls on higher‐level decision‐makers, and to bring wider attention to the likely health impacts of climate change—recognising, of course, that they are one part of a complex set of influences and considerations.^12^ This may be especially true if healthcare decision‐makers are calling for constraints on themselves.

Moreover, even if (or hopefully once) higher‐level decision makers take these allocation issues seriously, healthcare decision‐makers will not be without ways to make a difference. Higher‐level decision makers will need feedback on how any policies they institute are working, and how they affect healthcare allocation. Healthy systems have robust channels of communication and information flow between different levels of seniority, and they have broad alignment in the aims and goals across the system. This means that the system as a whole can be responsive to needs and changes. Thus, when it comes to environmental issues, especially those that affect health, healthcare decision makers ought to see themselves as important conduits between lower‐level and higher‐level decision‐making. Rather than take responsibility for environmental issues on their own shoulders, healthcare decision‐makers ought to use their position to identify and escalate problems. Instead of dealing with trade‐offs themselves, they should raise those trade‐offs at a higher level.

In this ‘feedback and implementation’ role, they also ought to take steps to ensure that higher‐level decisions are abided by in good faith. This means ensuring that any goals or targets do not simply become tick box exercises; that decision‐makers take honest appraisals, monitor progress, and avoid looking for gaps, loopholes, and so forth, in decision making. Whilst we advocate for higher‐level decision‐makers to institute side constraints, it is also important that healthcare allocators do not see these side‐constraints as impositions or barriers to their own goals. Environmental decision‐making ought not to be placed into the hands of healthcare decision‐makers, but they still need to be informed about why these decisions are being made and that they still have duties regarding the environment even though they are not responsible for weighing them themselves.

Nevertheless, it seems to us that two circumstances remain under which environmentally minded healthcare decision‐makers may act directly, and unilaterally, on environmental issues. The first is where there are direct, well‐evidenced, tractable health benefits to be had from certain interventions, then hospitals may pursue them, so long as they fit within the normal scope of their responsibilities. In such cases, the focus is on the health benefits, and they are assessed entirely on their health basis as per any other decision; the environmental benefit is either a positive externality—a nice addition—or merely the means by which the health benefit is achieved. For instance, as Joshua Parker argues, when it comes to asthma treatments there is already ‘a strong case for improving care by establishing patients on effective preventative treatments and reducing the reliance on short‐acting beta agonists’.^13^ In such circumstances, the environmental benefit of reducing GHG emissions is not necessary to justify a change in policy. Healthcare decision‐makers concerned about the environment would thus be permitted to search out these win‐win situations and act on them (of course following normal due process).^14^

Second, environmental issues may be sufficient to act as tiebreakers in cases between equally good options. Again, however, this is only because of their potential to benefit health. The health benefits of most environmental interventions are too uncertain and intractable to typically feature in our decisions, but when all other considerations are balanced, these intractable benefits can re‐enter the decision‐making process. For example, a hospital can choose the more environmentally friendly ambulance, if they cost the same and achieve the same job.

Importantly though, this only applies to goods which are largely commensurable. In the ambulance case, the direct and measurable health good of either option is equal, and we might realise some intractable health goods by reducing air pollution. However, when the goods are incommensurable, or a value trade‐off has to be made, then environmental issues ought not to be tiebreakers. For instance, suppose we can save person A or person B. Person B is further away, so sending an ambulance to him will produce more CO_2_. Nevertheless, the fact that saving A is the more environmentally friendly option (and thus mildly better for public health) is no tiebreaker. The identified lives are incommensurable and so environmental factors cannot be tiebreakers. As such, the scope of cases where environmental issues may be tiebreakers will be limited, and decision‐makers will need to be careful not to overstep the line.

Nonetheless, these cases may provide some easy healthcare‐related environmental wins. Healthcare decision makers can legitimately pursue broadly like for like replacements that also help to mitigate environmental damage. Yet we also need to be careful not to overstate the value of these wins; they should not be used to greenwash or get in the way of broader calls for systemic change. These interventions by themselves will not achieve environmental sustainability, and to ignore the cases where we must trade‐off between health care and the environment is to bury our heads in the sands. Whilst it is important not to let the hard questions get in the way of the easy wins, let us also not let the easy wins convince us we do not need to address the hard questions.

Finally, there is a deeper political issue, alluded to above, which underlies the arguments that we have presented in this paper. Responsibility for the environment and so, environmental sustainability is something that we all have as a collective, no matter what our occupations or circumstances are. But robust policymaking in this area is tough and requires the kind of strength and vision that those in power at the highest levels seemingly do not have. Consequently, there is an easy rhetoric which systematically devolves this responsibility to lower and lower levels: eventually, the collective responsibility is diluted to the set of responsibilities of individuals. This may be the consequence of a lazy kind of methodological individualism about moral responsibility, but we suspect that it might be more pernicious. None of this is to suggest that individuals, healthcare decision‐makers in particular, do not have obligations as citizens but as we have argued above, these are political obligations that are to be distinguished from their specific role responsibilities to distribute health care.

## Footnotes

1 It remains an open question whether representative democracies are the best way to settle this disagreement and provide legitimacy to these decisions or whether we should use more direct or deliberative democratic models to settle these particular problems. However, this issue sits outside the scope of our analysis, in practice this is the broadly accepted way that legitimacy is conferred to resource allocation decisions.

2 For a similar analysis of healthcare professional's environmental responsibilities see Lignou, S., & Hart, J. (2024). Navigating climate responsibility: A critical examination of healthcare professionals' moral duties. *Journal of Medical Ethics*, *50*, 376–377.

3 Daniels, N., & Sabin, J. E. (2002). Accountability for reasonableness. In N. Daniels & J. E. Sabin (Eds.), *Setting limits fairly: Can we learn to share medical resources?* (pp. 43–66). Oxford University Press; Daniels, N., Saltonstall, M. B., & Sabin, J. E. (2008). Accountability for reasonableness: An update. *BMJ*, *337*, a1850. https://doi.org/10.1136/bmj.a1850

4 Thames Valley Priorities Committee. (2021). *Ethical framework*; Sheehan, M., & Hope, T. (2012). Allocating health care resources in the UK: Putting principles into practice. In R. Rhodes, M. P. Battin, & A. Silvers (Eds.), *Medicine and social justice: Essays on the distribution of health care* (pp. 219–230). Oxford University Press.

5 DHSC & Public Health England. (2021). *Education, schooling and health summary*. UK Government. https://www.gov.uk/government/publications/education-schooling-and-health/education-schooling-and-health-summary#findings

6 McTernan, E. (2014). Should fertility treatment be state funded? *Journal of Applied Philosophy*, *32*, 227–240.

7 These problems indicate the importance of internal monitoring and feedback and communication across domains, in order to align systems through effective regulation, legislative side‐constraints, and proper incentives.

8 Of course, included with these vagaries are blurred boundaries between these different areas. It is not at all always clear when something stops being primarily a matter of health care and begins being a matter of education or social care. But the system functions well enough without sharp and arguably inappropriate distinctions. It would be a mistake to try to define ‘health’ in broad, aspirational terms—as we have suggested this would not match with the realities of our societal institutions and would inappropriately sacrifice depth for breadth.

9 The government may of course extend these requirements to non‐state funded organisations and to private individuals.

10 Joshua Parker's contribution to this edition goes into significantly more detail about what these thresholds might look like and how they could be justified.

11 It also seems likely that these approaches will be much more radical and much more robust, especially in light of actors within healthcare who might otherwise lack the motivation to care about the environment. There will be significantly less danger of greenwashing (i.e. interventions intended to make an institution look environmentally sustainable, but in reality make little or no real difference) or low impact interventions. These side constraints will also apply across the board, increasing consistency and fairness of application, and ensure that environmental issues do not become an afterthought, or a lower priority.

12 Wabnitz, K. J., Gabrysch, S., Guinto, R., Haines, A., Hermann, M., Howard, C., Potter, T., Prescott, S. L., & Redvers, N., (2020). A pledge for planetary health to unite health professionals in the Anthropocene. *The Lancet*, *396*, 1471–1473.

13 Parker, J. (2022). Barriers to green inhaler prescribing: ethical issues in environmentally sustainable clinical practice. *Journal of Medical Ethics*, *49*, 92–98. p. 93.

14 David Kirchhoffer and Bridget Pratt give several examples of win‐win situations in their contribution to this edition, which could make good starting points. However, whilst some of these interventions certainly seem to be win‐win in terms of improving health and the environment, on our view this will still be insufficient justification for pursuing them without higher‐level approval, if the cost is too high. For instance, the KTP hospital in Singapore that has been designed as a ‘biophillic’ hospital, may indeed improve both health and the environment. Yet we could imagine that the cost of such a hospital may be significant, such that the cost per QALY achieved is insufficient to justify it in purely health terms. These kinds of win‐wins would still be precluded from healthcare decision‐makers if they cannot be justified in purely health terms.

